# Bone Marrow Aspirate Concentrate for the Treatment of Avascular Meniscus Tears in a One-Step Procedure—Evaluation of an In Vivo Model

**DOI:** 10.3390/ijms20051120

**Published:** 2019-03-05

**Authors:** Matthias Koch, Selma Hammer, Julian Fuellerer, Siegmund Lang, Christian G. Pfeifer, Girish Pattappa, Johannes Weber, Markus Loibl, Michael Nerlich, Peter Angele, Johannes Zellner

**Affiliations:** 1 Department of Trauma Surgery, University Medical Centre Regensburg, Franz-Josef-Strauss-Allee 11, 93053 Regensburg, Germany; selma.hammer@stud.uni-regensburg.de (S.H.); julian.fuellerer@ukr.de (J.F.); lang.siegmund@gmail.com (S.L.); christian.pfeifer@ukr.de (C.G.P.); johannes1.weber@ukr.de (J.W.); markus.loibl@ukr.de (M.L.); michael.nerlich@ukr.de (M.N.); peter.angele@ukr.de (P.A.); johannes.zellner@ukr.de (J.Z.); 2 Laboratory of Experimental Trauma Surgery, Department of Trauma Surgery, University Medical Centre Regensburg, Franz-Josef-Strauss-Allee 11, 93053 Regensburg, Germany; girish.pattappa@ukr.de; 3 Sporthopaedicum Regensburg/Straubing, Hildegard-von-Bingen-Str. 1, 93053, Regensburg, Germany

**Keywords:** meniscus, avascular meniscus, meniscus regeneration, BMAC, PRP

## Abstract

Avascular meniscus tears show poor intrinsic regenerative potential. Thus, lesions within this area predispose the patient to developing knee osteoarthritis. Current research focuses on regenerative approaches using growth factors or mesenchymal stem cells (MSCs) to enhance healing capacity within the avascular meniscus zone. The use of MSCs especially as progenitor cells and a source of growth factors has shown promising results. However, present studies use bone-marrow-derived BMSCs in a two-step procedure, which is limiting the transfer in clinical praxis. So, the aim of this study was to evaluate a one-step procedure using bone marrow aspirate concentrate (BMAC), containing BMSCs, for inducing the regeneration of avascular meniscus lesions. Longitudinal meniscus tears of 4 mm in size of the lateral New Zealand White rabbit meniscus were treated with clotted autologous PRP (platelet-rich plasma) or BMAC and a meniscus suture or a meniscus suture alone. Menisci were harvested at 6 and 12 weeks after initial surgery. Macroscopical and histological evaluation was performed according to an established Meniscus Scoring System. BMAC significantly enhanced regeneration of the meniscus lesions in a time-dependent manner and in comparison to the PRP and control groups, where no healing could be observed. Treatment of avascular meniscus lesions with BMAC and meniscus suturing seems to be a promising approach to promote meniscus regeneration in the avascular zone using a one-step procedure.

## 1. Introduction

An intact meniscus is essential to a healthy knee joint [1,2]. Any loss of meniscus integrity is associated with a loss of function, such as load bearing, shock absorption, stabilization, or proprioception of the knee joint, and subsequent biomechanical derangement [3,4,5,6,7,8]. The increased load to the articular cartilage predisposes patients to the development of osteoarthritic changes [9,10,11,12,13].

Based on this knowledge, previously developed regenerative-medicine-based meniscus therapy options have focused on successful repair strategies, such as meniscus suturing that has been established in the clinical routine for peripheral meniscus lesions in the vascular area [14,15]. However, due to the poor intrinsic regenerative potential in the avascular meniscus areas, partial meniscectomy still remains the main treatment option in meniscus surgery [16,17,18]. The current literature describes new therapeutic approaches, especially for meniscus lesions in the avascular zone. Included within this discussion are the regenerative effects of biologic agents, such as platelet-rich plasma (PRP) or mesenchymal stem cells (MSCs) [12,16,18,19,20,21,22]. Whilst the impact of PRP is, at present, controversial, the beneficial effect of MSCs for meniscus regeneration has gained approval [18,21,22,23]. However, clinical translation of either of these repair approaches has yet to be successfully established. The main problems for the clinical use of MSCs are the regulatory burdens, high costs, and the complex harvest, preparation, and application procedures in a two-step procedure. The Angel System (Arthrex, Naples, FL) is a commercially available and fully automated blood and bone marrow processing system that has already been used in orthopedic surgery for the preparation of PRP and MSC-containing bone marrow aspirate concentrate (BMAC) [24]. Therefore, the aim of this study was to evaluate meniscus regeneration within avascular meniscus tears after the application of PRP and BMAC in combination with a meniscus suture in a one-step procedure.

## 2. Results

### 2.1. Meniscus Suture with PRP—6 Weeks

**Figure 1:**
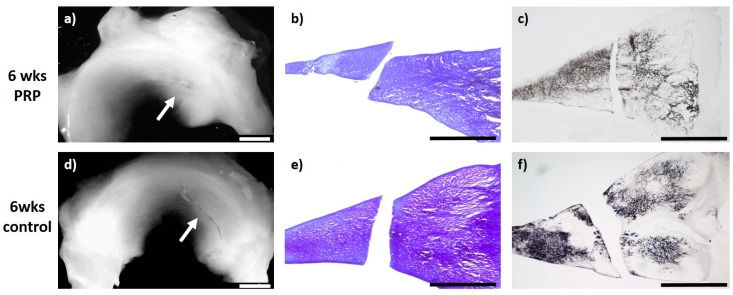
(**a**) + (**d**): Macroscopic view of a lateral meniscus of New Zealand White rabbits. The created meniscus tears in the avascular parts of the pars intermedia are still visible in the control (**d**) as well as in the platelet-rich plasma (PRP)–meniscus suture group (**a**) after a study period of 6 weeks, each marked by the white arrow. (**b**) + (**e**): Microscopic view (4× enlargement), dimethylmethylen blue (DMMB) staining of the lateral meniscus 6 weeks after surgery. No healing was seen in the control (**e**) or in the PRP–meniscus suture group (**b**). (**c**) + (**f**): Microscopic view (4× enlargement). Collagen type II staining of the lateral meniscus 6 weeks after surgery. No healing was seen in the control (**f**) or in the PRP–meniscus suture group (**c**). Benchmark: bar (white) = 2 mm, bar (black) = 1 mm.

The evaluation of the menisci after meniscus therapy with intralesional PRP application and meniscus suture and after a study period of 6 weeks (*n* = 6) macroscopically showed no meniscus healing and a remaining meniscus tear. Microscopically, in two menisci, the slightest signs of a healing reaction were observed unilaterally containing no cells and with slight staining for proteoglycan or collagen type II. Signs of inflammation or foreign body reaction were found in none of the animals (see Figure 1).

### 2.2. Meniscus Suture with BMAC—6 Weeks

**Figure 2:**
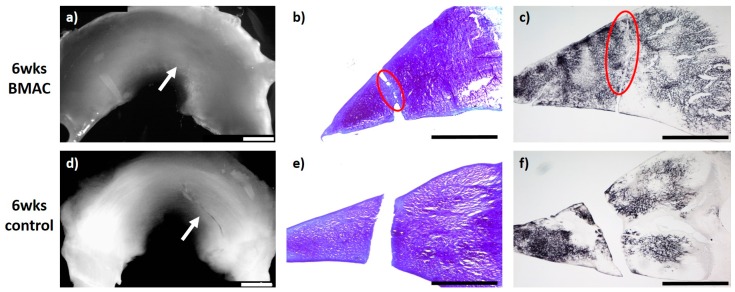
(**a**) + (**d**): Macroscopic view of a lateral meniscus of New Zealand White rabbits. The created meniscus tears in the avascular parts of the pars intermedia are still visible in the control marked by the white arrow (**d**). In the bone marrow aspirate concentrate (BMAC)–meniscus suture group, macroscopically visible meniscus healing was found after 6 weeks, marked by the white arrow. (**a**). (**b**) + (**e**): Microscopic view (4× enlargement), DMMB staining of the lateral meniscus 6 weeks after surgery. No healing was seen in the control (**e**). Partial defect filling was observed 6 weeks after BMAC–meniscus suture treatment, marked with a red circle (**b**). (**c**) + (**f**): Microscopic view (4× enlargement). Collagen type II staining of the lateral meniscus 6 weeks after surgery. No healing was seen in the control (**f**). Partial defect filling was observed 6 weeks after BMAC–meniscus suture treatment as marked with a red circle (**c**). Benchmark: bar (white) = 2 mm, bar (black) = 1 mm.

In the group of meniscal tears treated with BMAC and meniscus suture (*n* = 6), in five animals, a defect-filling tissue, unilaterally or bilaterally partial integrated, was observed. In these menisci, the surface of the meniscus tissue was ruptured. Overall, just a few cells without a meniscus-like appearance were found in the repair tissue. Both the type II collagen content and the content of proteoglycans appeared generally moderate. The newly developed tissue of three animals was stable in shape. In the remaining three animals, the repair tissue was of poor quality and ruptured before stability testing, wherein one of these menisci showed no defect filling or regeneration reaction. Signs of inflammation or foreign body reaction were found in none of the animals (see Figure 2).

### 2.3. Meniscus Suture with PRP—12 Weeks

**Figure 3:**
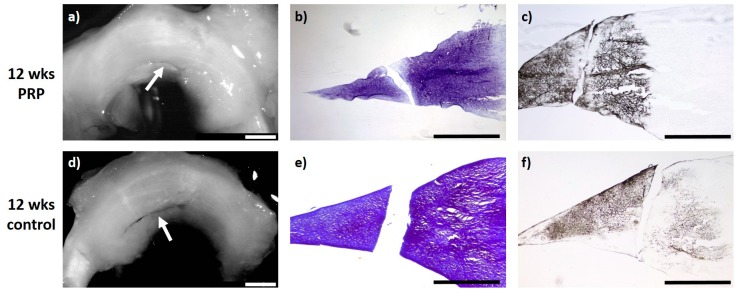
(**a**) + (**d**): Macroscopic view of a lateral meniscus of New Zealand White rabbits. The created meniscus tears in the avascular parts of the pars intermedia are still visible in the control (**d**) as well as the PRP–meniscus suture group (**a**) after a study period of 12 weeks, each marked by the white arrow. (**b**) + (**e**): Microscopic view (4× enlargement), DMMB staining of the lateral meniscus 12 weeks after surgery. No healing was seen in the control (**e**) or in the PRP–meniscus suture group (**b**). (**c**) + (**f**): Microscopic view (4× enlargement). Collagen type II staining of the lateral meniscus 12 weeks after surgery. No healing was seen in the control (**f**) or in the PRP–meniscus suture group (**c**). Benchmark: bar (white) = 2 mm, bar (black) = 1 mm.

Regarding intralesional PRP application and meniscus suture of meniscus tears after a study period of 12 weeks, all evaluated menisci (*n* = 6) macroscopically showed no meniscus healing with an obviously remaining meniscus tear. The microscopic evaluation showed a marginal unilateral healing reaction in two menisci containing no cells and with a slight staining for proteoglycan or collagen type II. None of the animals showed signs of inflammation or foreign body reaction (see Figure 3).

### 2.4. Meniscus Suture with BMAC—12 Weeks

**Figure 4:**
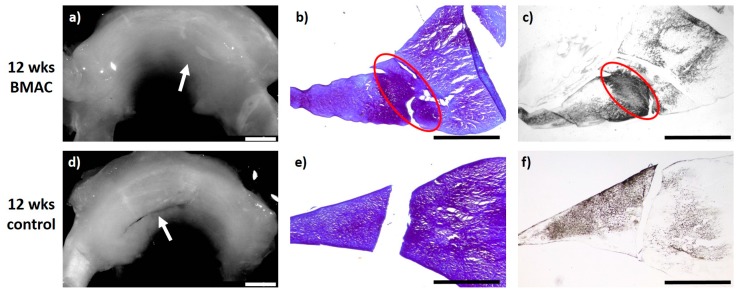
(**a**) + (**d**): Macroscopic view of a lateral meniscus of New Zealand White rabbits. The created meniscus tears in the avascular parts of the pars intermedia are still visible in the control (**d**), marked by the white arrow. In the BMAC–meniscus suture group, macroscopically visible meniscus healing was found after 12 weeks (**a**), marked by the white arrow. (**b**) + (**e**): Microscopic view (4× enlargement), DMMB staining of the lateral meniscus 12 weeks after surgery. No healing was seen in the control (**e**). Almost full defect filling with bilateral integration was found 12 weeks after BMAC application and meniscus suture, marked with a red circle (**b**). (**c**) + (**f**): Microscopic view (4× enlargement). Collagen type II staining of the lateral meniscus 12 weeks after surgery. No healing was seen in the control (**f**). Almost full defect filling with bilateral integration and clear collagen type II staining was found 12 weeks after BMAC application and meniscus suture, marked with a red circle (**c**). Benchmark: bar (white) = 2 mm, bar (black) = 1 mm.

Twelve weeks after meniscus defect treatment with BMAC and meniscus suturing (*n* = 6), an almost complete defect filling was achieved in three cases, while in two animals the defect filling was more than one-half complete. In one meniscus, a macroscopically unstable and microscopically reduced healing reaction was observed. In three menisci, the surface of the meniscus tissue appeared fissured, whereas in the other three cases the surface was found to be ruptured. A complete integration into the surrounding native meniscus was reached in one case, while unilateral complete or bilateral partial integration was observed in three menisci. Two animals presented with partial unilateral integration. The repair tissue of five menisci was infiltrated by cells of a meniscus-like type and with a clear staining for proteoglycan. The collagen type II content was moderate to high in the regenerated tissue of three menisci. In the regenerated tissue of the remaining three menisci, a low staining intensity for collagen type II was found. The repair tissue was stable to pressure and pulling stress in one animal and also stable in shape in two cases, whilst in the remaining three animals, it appeared weak. None of the animals showed signs of inflammation or foreign body reaction (see Figure 4).

### 2.5. Meniscus Scoring

**Figure 5:**
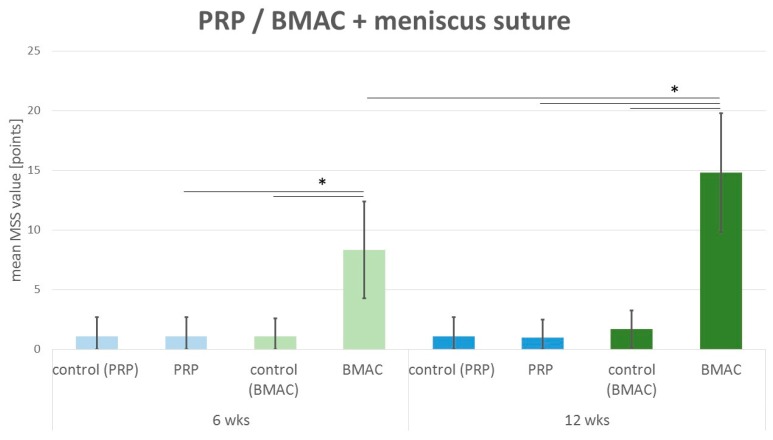
Mean meniscus scoring value: comparison of the meniscus scoring system (MSS) results of the control group with isolated meniscus suture (denoted control) and the PRP group with intralesional PRP application and meniscus suture (PRP) after 6 weeks (6 wks) and 12 weeks (12 wks) as well as the BMAC group with intralesional BMAC application and meniscus suture (BMAC) after 6 weeks (6 wks) and 12 weeks (12 wks). Significant differences (*p* < 0.05) are indicated by a bar and *.

After six weeks, the meniscus scoring system showed no meniscus regeneration in the control (mean 1.1 points; SD 1.6 points). In the PRP–meniscus suture group (mean 1.1 points; SD 1.6 points), no meniscus regeneration was detected (see Figure 5). In comparison to these results, 6 weeks after intralesional BMAC application and meniscus suture, a significant tendency to meniscus healing was detected (mean 8.3 points; SD 4.1 points; *p*(6 weeks BMAC vs. 6 weeks control) = 0.005; *p*(6 weeks BMAC vs. 6 weeks PRP) = 0.001) (see Figure 5). 

Even after 12 weeks, no regenerative reaction to meniscus therapy was described in the control group with an isolated meniscus suture (mean 1.1 points; SD 1.6 points) or in the PRP–meniscus suture group (mean 1.0 points; SD 1.5) (see Figure 5). Concerning the treatment with BMAC and meniscus suture (mean 14.8 points; SD 5.0 points), significantly enhanced meniscus regeneration was found in comparison to the 12 week control (*p* = 0.002) and 12 week PRP–meniscus suture group (*p* < 0.001) and in comparison to the 6 week BMAC–meniscus suture group (*p* = 0.002) (see Figure 5). 

## 3. Discussion

In this study, it was shown that meniscus regeneration in meniscus tears located in the avascular and partial vascular area can be positively influenced within a one-step procedure by the application of BMAC and meniscus suture in a New Zealand White Rabbit model. After six weeks, the BMAC–meniscus suture approach showed significant advantages in treating avascular tears as described by the Meniscus Scoring System in comparison to the PRP–meniscus suture and the isolated meniscus suture group (control). The beneficial effect on meniscus regeneration was enhanced 12 weeks after the index procedure in comparison to the MSS values in the 6 week BMAC–meniscus suture group and those in the 12 week PRP–meniscus suture group and control. Thus, BMAC-augmented meniscus suturing offers new treatment modalities for meniscus repair of avascular and partial vascular meniscus lesions.

BMAC is also an easily available autologous cell source in a clinical situation. It contains concentrated multipotent stem cells and growth factors [25,26,27,28,29]. As progenitor cells, MSCs are able to enhance tissue regeneration in a multifactorial way [16,22,30,31,32,33], either by direct differentiation into repair cells according to the surrounding tissue [16,22] or in an indirect way by the release of bioactive mediators, such as cytokines and growth factors, inducing both paracrine and autocrine activities [30,31]. Through this indirect mode of action, vascularization, amongst other modalities, is known to positively affect meniscus regeneration. By special mediators, survival and maturation of vascular cells and the stimulation of neovascularization, i.e., via the release of vascular endothelial growth factors (VEGF), are increased [31,34]. As an example, Tang et al. implanted MSCs into an ischemic area of cardiac muscle tissue after myocardial infarction and found increased VEGF level, vascular density, and perfusion grade, whilst the rate of apoptosis was concomitantly reduced due to the secretion of bioactive mediators [35] and the direct conversion of MSCs into endothelial cells [16,36]. Regarding the current literature, there is also much evidence of a beneficial effect of MSCs on meniscus regeneration [18,32,37,38]. Murphy et al. described a similar effect investigating fractional regeneration of lost meniscus tissue after partial meniscectomy with vascularization of the neo-meniscus after application of hyaluronic acid in combination with labelled MSCs and detection of these cells in the repair tissue [37]. In addition to the mentioned vascularization influencing effect, Horie et al. found enhanced expression of collagen type II after intra-articular MSC injection in rats after partial meniscectomy [39]. This finding is in accordance with the observations in the present study. Twelve weeks after BMAC application in avascular meniscus lesions, clear staining for collagen type II, as a marker for meniscal tissue remodeling within the inner zone, was detected [40,41]. 

There are further studies investigating the effect of MSCs in the context of meniscus repair in avascular and partial vascular meniscus lesions in acute meniscal lesions as well as in an early osteoarthritis situation [20,21,38,42]. Overall, all these studies showed promising results. Nevertheless, regarding the details, in all of these studies, MSCs were implanted in a two-step procedure that was complex in terms of harvesting, isolation, and culturing procedures and preparation—for example, the pre-seeding of carriers such as scaffolds before implantation. This is generally associated with extended procedures and high costs and particularly increases the regulatory burden for clinical translation. In contrast to that, the use of BMAC offers the opportunity to harvest, process, and implant progenitor cells with multilineage differentiation potential in a one-step procedure, whilst respecting the regulatory criteria [28,29]. Due to this, it is currently approved by the United States Food and Drug Administration (FDA) [28,29,43]. Relevant disadvantages in comparison to the regenerative quality provided by the implantation of isolated MSCs in a two-step procedure do not seem to exist. Zellner et al. [20] investigated the effect of hyaluronan collagen-based scaffolds loaded with MSCs, produced in a two-step procedure, for the treatment of avascular meniscus tears in a rabbit model. They also found a significant beneficial effect from the use of MSCs. However, the evaluation according to the MSS showed results comparable to the MSS values in the present study. So, it can be assumed that BMAC has a similar regenerative quality concerning meniscus regeneration compared to MSCs, despite the low percentage of MSCs within BMAC (0.001–0.01%) as described in previous literature [28,43]. By centrifugation of the bone marrow, the cellular components are in distinctly different layers with mononucleated cells, such as white blood cells, MSCs, hematopoietic stem cells, and platelets, separated from red blood cells [28].

Regarding the results of this study, and in summary of the current literature, the presence of progenitor cell populations such as MSCs seems to be essential for meniscus regeneration. Even though there are few publications documenting the beneficial effect of isolated PRP application for meniscus repair [3,44,45,46,47], many studies also investigating this issue were not able to prove a significant benefit of isolated growth factor application, e.g., use of single-use PRP [20,21,22,48,49,50]. This emphasizes the results of the present study. In both PRP groups, after 6 and 12 weeks, no trend of meniscus regeneration in comparison to the control could be detected.

However, there are many data supporting the combined use of MSCs and PRP as a source of MSCs and stimulating growth factors [51,52,53,54,55]. The presence of PRP-released growth factors enhances the proliferation capacity of MSCs without modifying their differentiation potential [52,53]. Further studies found that transforming growth factor (TGF-β), as one of the main growth factors released by PRP, controls migratory meniscus progenitor cells located in the avascular meniscus [56] and is involved in the induction of MSC differentiation towards a meniscus cell phenotype [57]. BMAC comprises both MSCs and platelets. Regarding the current literature, the percentage of MSCs in BMAC is described as being between 0.001 and 0.01% [28] and the percentage of platelets is increased up to 2.5-fold in comparison to that in regular PRP [27]. Thus, the combined regenerative effect of MSCs and the regeneration boosting effect of the platelets might explain the comparable results between the MSS values of the BMAC–meniscus suture group and the results of Zellner et al. [20] even if the amount of MSCs in a two-step procedure will surely be increased. Promising techniques and results concerning the successful regenerative use of BMAC in orthopedic surgery have already been introduced for shoulder, spine, and bone healing issues [24,58,59]. However, to the authors’ knowledge, there is no information concerning meniscus regeneration. Thus, the present study describes for the first time the beneficial use of BMAC for avascular meniscus tear regeneration in a one-step procedure.

Nevertheless, there are a few limitations standing in contrast to the strength of the present study. Even though the New Zealand White rabbits are an established model for meniscus research [12,20,21,22], the small size of the rabbit meniscus might limit the surgical procedure and precision. Additionally, due to the animal size, bone marrow harvest was technically limited to 20 mL. Therefore, peripheral blood was added to reach the required 40 mL volume for BMAC processing by the Angel System. Based on this, the harvested BMAC volume was reduced so much that BMAC was used for surgery and no further BMAC for analysis and characterization was available. Based on the clinical routine of rehabilitation after tissue injuries, the experiments were terminated after investigation periods of 6 and 12 weeks without a longer observation period. Furthermore, even if there was no technical change, the BMAC autologous thrombin mix demonstrated heterogeneous clotting properties that were also reflected in the SD of the MSS analysis. Thus, further studies using BMAC analysis and a reliable BMAC fixation technique in a larger experimental animal model and a longer investigation period are required before clinical translation.

## 4. Material and Methods

The local government’s animal rights protection authorities approved this study in accordance with the National Institutes of Health guidelines for the use of laboratory animals (Regierung von Unterfranken; 55.2DMS-2532-2-127, date of approval: 30.08.2015).

### 4.1. Study Design

Twenty-four male New Zealand White rabbits (aged 12–14 weeks and weighing 2.8–3.2 kg) were used in this study. All included rabbits received the treatment on the right knee joint and the control on the left knee joint. Two groups were differentiated according to the study period of 6 or 12 weeks. Each group consisted of six animals (Table 1). All animals were kept in single-animal cages in an air-conditioned environment and a 12 h/12 h day/night rhythm for the whole study period with free access to food and water.

### 4.2. Bone Marrow Harvest and Bone Marrow Aspirate Concentrate Preparation

For preparation of the BMAC, bone marrow was harvested immediately before surgery. New Zealand White rabbits were anesthetized using a combined intramuscular application of 0.6 mL/kg of ketamine 10% and xylazin 2%. Bone marrow was harvested by puncture of the iliac crest of the rabbits two times on each side by a small incision and penetration of the bone cortex with an 18-gauge needle. Afterwards, the penetration needle was changed to a heparin-flushed (1000 IU/mL) 18-gauge needle and the bone marrow was collected in a heparin-flushed (1000 IU/mL) 5 mL syringe containing 0.5 mL acid citrate dextrose anticoagulant (ACD-A). Overall, by four punctures, a total volume of 20 mL bone marrow aspirate (BMA) (4 × 5.5 mL including 4 × 0.5 mL ACD-A) was harvested. The BMA volume was limited to 20 mL due to the animal size. To obtain the minimum volume of 40 mL required for the processing system, 20 mL autologous whole blood harvested as described in Section 4.3 was added. The BMA–whole blood mix was then transferred to the Angel System (Arthrex, Naples, FL, USA) (a clinically established routine) automated system used for the sterile PRP and BMAC processing. A 7% hematocrit setting was used to obtain the maximum stem cell concentration. Autologous thrombin was prepared from platelet-poor plasma, a residue product of the process of obtaining BMAC, by an additional activAT system (Arthrex, Naples, FL). Before implantation in a sterile setting, 40 µL autologous thrombin was added to 360 µL BMAC to induce a clot. Clot formation was achieved by activation of the platelets included in the BMAC and subsequent initiation of fibrin matrix formation. Overall, the clotting properties of the BMAC–autologous thrombin mix were heterogeneous. The clot qualities differed with regard to their stiffness and the start of the clotting process.

During the BMA processing (approximately 20 min) and autologous thrombin preparation (approximately 20 min), surgery was performed as described in Section 4.4.

### 4.3. Harvest of Platelet-Rich Plasma and Preparation

For PRP preparation, autologous whole blood was harvested immediately before the index surgery. The New Zealand White rabbits were anesthetized using a combined intramuscular application of 0.6 mL/kg of ketamine 10% and xylazin 2%. Blood was harvested by cannulation of the ear artery of the rabbits with a 22-gauge needle. Blood was taken with a 5 mL syringe containing 0.5 mL ACD-A. This process was repeated with subsequent 5 mL syringes until a total volume of 40 mL of whole blood was obtained. The blood was then transferred to the Angel System (Arthrex, Naples, FL) and the 2% hematocrit setting was used. Autologous thrombin was prepared as described above, and prior to implantation in a sterile setting, 40 µL autologous thrombin was added to 360 µL PRP to induce a clot. The clotting process started in all samples after a mean of approximately one minute and showed homogeneous clotting properties.

During the PRP processing (approximately 20 min) and autologous thrombin preparation (approximately 20 min), surgery was performed as described below.

### 4.4. Surgical Procedure for Meniscal Tears

**Figure 6:**
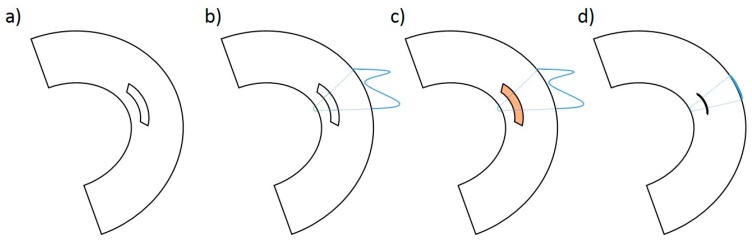
Schematic image of the defect preparation in the avascular area of the lateral meniscus (**a**), preparation of the meniscus suture in a modified “outside-in” technique (**b**), application of PRP (orange marked) or BMAC (orange marked) in the case of the treatment group (**c**), and meniscus suture with reduction of the meniscus defect (**d**).

Surgery was performed bilaterally in 24 New Zealand White rabbits by an experienced orthopedic surgeon. The rabbits were anesthetized using a combined intramuscular application of 0.6 mL/kg of ketamine 10% and xylazin 2% before BMA and PRP harvest. During BMAC and PRP preparation, the surgical procedure to create avascular meniscus tears began with the control on the left side and, subsequently, the index surgery on the right side. Meniscus exposure was achieved by a lateral parapatellar approach. The lateral meniscus was luxated anteriorly by a limited soft tissue release. In the avascular area of the pars intermedia, a longitudinal meniscus tear (approximately 4 mm long) was created using a stitch scalpel (Feather disposable scalpel Nr.11, Osaka, Japan) (see Figure 6a). The meniscus lesions were treated according to the different experimental groups (Table 1) (see Figure 6: treatment group: (a) − (d); control group: (a) + (b) + (d)). Following treatment, the meniscus tears were sutured in an outside-in technique with a resorbable 6-0 PDS suture (see Figure 6d). After reduction of the meniscus, the capsule was reattached with 3-0 resorbable sutures in a continuous suture pattern and skin closure was achieved with a resorbable running subcuticular suture. Wound healing was checked daily. Postoperative pain control was achieved by subcutaneous application of carprofen 5 mg/kg. There were no limitations concerning postoperative movement and weight bearing. The animals were euthanized at 6 or 12 weeks by an intravenous application of an overdose of narcoren (0.5 g/kg). The New Zealand White rabbits were anesthetized using a combined intramuscular application of 0.6 mL/kg of ketamine 10% and xylazin 2% beforehand. [12]

### 4.5. Assessment of the Menisci

Gross assessment of joint morphology was performed as previously described by Zellner et al. [20,22]. Rabbits were sacrificed in deep narcosis, as used for surgery, by an overdose of intravenously administered narcoren either 6 or 12 weeks after index surgery, depending upon the groups. The menisci were harvested after exposure of the knee joints. Afterwards, the macroscopic morphology of the meniscus was evaluated and photographed. The correct anatomic location of the menisci, the macroscopic integration of the repair tissue, the state of the meniscus surface, and the color changes were evaluated. All menisci were analyzed by two experienced and blinded scorers and the results collected using an established scoring system as described below. Afterwards, the lateral menisci were harvested for further histological examination and photographic documentation, as previously described [12,20,22].

### 4.6. Histology

The lateral menisci harvested from the in vivo experiments were fixed in a solution containing 4% paraformaldehyde and 15% picric acid, embedded in Tissue-Tek OCT (Sakura Finetek, Tokyo, Japan) and frozen in liquid nitrogen. All samples were cut in 10 µm transversal sections and every tenth one of them was stained with dimethylmethylen blue (DMMB) to determine the content of proteoglycan.

Overall, two blinded scorers, both knowledgeable in the knee anatomy of rabbits and in histological assessment, analyzed the sections according to the established scoring system. [6,12]

### 4.7. Immunohistochemistry

#### Type II Collagen

For immunohistochemical analysis, frozen sections were prepared as previously described [6,12,21]. Embedded in Tissue-Tek OCT (Sakura Finetek, Tokyo, Japan), the samples were washed in phosphate-buffered saline and subsequently digested with 0.1 % pepsin at pH 3.5 for 15 min to facilitate antibody access to the target epitopes. Type II collagen was immunolocalized by the immunoperoxidase ABC technique (Vector, Burlingame, CA, USA). Anti collagen II (clone II-4C11; Calbiochem Merck, Schwalbach Germany) was used as primary antibody with an antibody dilution of 1:100. After staining with biotin-conjugated polyclonal goat anti-mouse IgG secondary antibody (Jackson, West Grove, PA, USA), positive signals were visualized using nickel- and cobalt-enhanced 3,3’-diaminobenzidine (DAB).

### 4.8. Meniscus Scoring System

Evaluation of meniscus regeneration was performed according to an established and validated scoring system for the analysis of repair tissue in meniscal tears previously published by Zellner et al. [20,21].

Macroscopical analysis included the evaluation of stability and defect filling with repair tissue. Stability was quantified according to the following levels: no stability in the case of no macroscopically visible meniscus healing before the harvest of the meniscus; weak stability in the case of macroscopically visible signs of meniscus healing before harvest, but no macroscopically visible meniscus healing during harvest; and stable in shape if there were macroscopically visible signs of meniscus healing during the harvest process but no microscopically visible meniscus healing, or stable to pressure and pulling stress if microscopically visible meniscus healing was detected. The other categories were the microscopical assessment of the quality of the surface area, the integration of the repair tissue to the surrounding native meniscus, cellularity, cell morphology, and proteoglycan and collagen II content. The assessment involved eight individual scoring subgroups evaluating meniscus regeneration, each receiving a scoring value ranging from 0 (no repair) to 3 (meniscus-like tissue). The values of these items were summed up, consequently reaching a combined score from 0 (no repair) to 24 (complete reconstitution of the meniscus) (Table 2). Two experienced blinded scorers conducted the data collection. A high internal consistency has been attributed to this scoring system (Cronbach’s α = 0.88) [20]. Therefore, study results can be easily interpreted and compared to those of other studies using this score.

### 4.9. Statistical Analysis

Statistical analysis was performed using SPSS software version 23.0 (SPSS, Chicago, IL, USA). For determining whether the data followed a Gaussian distribution, a Kolmogorov–Smirnov test was performed. For intra-group comparison, a Wilcoxon test was used. For inter-group comparison, the Mann–Whitney *U*-test was chosen. A probability value of less than 0.05 was set as the level of statistical significance for all evaluations.

## 5. Conclusions

The intralesional application of autologous BMAC in addition to a meniscus suture resulted in successful meniscus regeneration of tears in the avascular zone by a one-step procedure. In contrast, treatment with intralesional PRP augmentation of meniscal sutures showed no impact on meniscus regeneration of avascular meniscus tears. For potential clinical translation, BMAC seems to be a promising approach for the biological augmentation of sutures in critical meniscal areas to enhance healing capacity in the avascular zone. 

## Figures and Tables

**Table 1:** Experimental groups.

Group	Treatment			6 Weeks	12 Weeks
PRP	meniscus suture	+	clotted PRP	6 animals, right knee	6 animals, right knee
BMAC			clotted BMAC	6 animals, right knee	6 animals, right knee
control			−	each contralateral knee	each contralateral knee

The minus means that no additional treatment was added to the meniscus suture.

**Table 2:** Meniscus scoring system for the evaluation of meniscus repair tissue [12,20,21].

Scoring Subgroup	0	1	2	3
Defect filling	No fill	<25%	25–75%	>75%
Surface	No surface	Ruptured	Fissured/fibrillated	Meniscus-like
Integration	No integration	Partial, unilateral integration	Bilateral partial or unilateral complete integration	Bilateral complete integration
Cellularity	No cells	>10 Cell clusters/slide	No cell clusters/slide, Cell/ECM ratio >0.5	Meniscus-like cell/ECM ratio
Cell morphology	No cells	<25% Meniscus-like cells	25–75% Meniscus-like cells	>75% Meniscus-like cells
Content of proteoglycan	No staining for proteoglycan	<25%	25–75%	>75%
Content of collagen II	No staining for collagen II	<25%	25–75%	>75%
Stability	No stability	Weak	Stable in shape	Stable to pressure and pulling stress

ECM = extracellular matrix.
